# Fatty acid profiling of 75 Indian snack samples highlights overall low trans fatty acid content with high polyunsaturated fatty acid content in some samples

**DOI:** 10.1371/journal.pone.0225798

**Published:** 2019-12-05

**Authors:** Kshamata Joshee, Tanvi Abhang, Ram Kulkarni

**Affiliations:** Symbiosis School of Biological Sciences, Symbiosis International (Deemed University), Lavale, Pune, India; Universitat Wien, AUSTRIA

## Abstract

Diet-derived fatty acids have well-proven varying effects on human health. In particular, trans fatty acids (TFA) are associated with high risk of cardiovascular diseases whereas, polyunsaturated fatty acids (PUFA) are considered to be beneficial to the human health. In this study, we report fatty acid profiling of 75 food samples from India belonging to three broad categories, viz., perishable deep-fried, non-perishable deep-fried and bakery. Lipids were extracted from the snacks and fatty acids converted into methyl esters and analysed by gas chromatography. Thirty-seven detected fatty acids were classified into four categories: saturated (SFA), monounsaturated (MUFA), PUFA, and TFA, of which SFA represented the most abundant class in two-third of the samples. The highest average proportions of TFA and SFA of 3.26% and 56.1%, respectively, in total fatty acids were found in the bakery products; whereas, that of PUFA (38%) in the perishable deep-fried products. Principal Component Analysis depicted clustering of many samples according to the above-mentioned categories and helped predict the oil usage. Lower TFA content in all the samples and high proportion of PUFA in a quarter of the samples is suggestive of a better trend as compared to earlier studies.

## Introduction

Fatty acids originating from the diet have varying effects on the cardiovascular health of humans. Poly- and mono-unsaturated fatty acids (PUFA and MUFA) generally reduce the risk of cardiovascular diseases (CVD); saturated fatty acids (SFA) are modestly positively correlated with the risk of CVD; whereas, trans fatty acids (TFA) have well-proven strong association with the high risk of CVD [1]. Thus, monitoring the fatty acid composition of foods is an important process which can help maintain healthy life-style.

Majority of such TFA are generated during the processing and usage of the natural oils. During industrial hydrogenation of the vegetable oils, apart from getting converted into saturated derivatives, some of the unsaturated fatty acids are also converted into trans isomeric forms [2]. In addition, deodorization and repeated usage of vegetable oils for frying is also reported to produce trans fatty acids [3,4].

Small-scale food entrepreneurs, especially in the developing countries like India, are always under financial burden. This forces those to follow some cost-cutting practices such as repeated usage of the oils for frying, usage of hydrogenated fats to lengthen the shelf-life of the fried material and adulteration in the oils used for preparing the foods [4,5]. Several problems associated with the fat quality in the Indian food supply further worsen the situation [6]. Foods prepared through such practices are scarcely inspected for the fatty acid composition. India, the second highly populated country in the world with enormous diversity in the food products and culinary practices, represents an ideal place for examining the fatty acid profiles of the commonly consumed snacks. A very few studies have been carried out in India for determining the fatty acid composition of the commonly consumed snacks [5,7,8]. In this study, we report fatty acid profiling of 75 samples of commonly consumed snacks collected from 13 towns. The analysis suggests high variability in the fatty acid profiles with relatively low proportion of trans fatty acids.

## Materials and methods

### Fatty acid extraction and hydrolysis

Food samples were collected from various places in India as detailed in S1 Table during November 2017-February 2018 and stored at -20°C for a maximum period of 25 days until further analysis. The selection of food samples was based on general popularity in India as well as availability. Extraction, derivatization and analysis of the fatty acids was based on the Association of Official Analytical Chemists (AOAC) Official Method 996.06 [9]. The samples were finely homogenized using a homogenizer (IKA A11 basic analytical mill) prior to fat extraction. To an accurately weighed 0.25 g homogenized food sample, 25 mg of pyrogallic acid and 0.5 mL of a solution containing 2.5 mg triundecanoin (internal standard) in chloroform were added. To this mixture, 0.5 mL of ethanol was added and the entire product was dispersed in 2.5 mL of 8.3 M HCl by brief vortexing. The vials were incubated in a water bath at 80°C for 40 min with intermittent shaking. Five millilitres each of diethyl ether and petroleum ether were added with vortexing between the additions. The ether extract was decanted into a 50 mL beaker after centrifugation, and evaporated to dryness using nitrogen stream.

### Methyl esterification

To the solvent-free fats, 0.5 mL of 10% boron trifluoride methanol complex solution and 0.25 mL toluene were added and incubated at 100°C for 1 h with shaking every 10 min. After cooling to room temperature, 2 mL deionized water, 1.5 mL hexane and 0.25 g sodium sulphate were added and tubes were shaken. After phase separation, the top layer was dried with sodium sulphate and transferred into a GC vial.

### Gas chromatographic analysis

Analyses of FAMEs was carried out on GC-FID (Shimadzu GC 2014) equipped with Trace TR-FAME GC column (Thermo Scientific) having the dimensions of 120 m x 0.25 mm x 0.25 μm. Nitrogen was used as a carrier gas with flow of 0.75 ml min^-1^. The initial oven temperature was 100°C for 4 minutes; which was raised to 240°C at the ramp of 3°C min^-1^ and held for 30 min. Injector and detector ports were maintained at 225°C and 285°C, respectively. A microliter sample was injected using autosampler with split ratio of 1:50. Authentic standards of FAMEs were procured from Sigma and used for identification of the FAMEs in the samples by matching the retention times. This include Supelco 37 component mix (CRM47885), linoleic acid methyl ester isomer mix (CRM47791), linolenic acid methyl ester isomer mix (CRM47792), FAME mix unsaturates C20:1-C20:5 (18912), methyl undecanoate (U0250), methyl arachidate (A3881), methyl cis-13 docosenoate (45659), methyl heneicosanoate (H3265), methyl tricosanoate (T9900), methyl tetracosanoate (L6766), cis-13,16-docosadienoic acid methyl ester (D4034), and cis 11,14,17 eicosatrienoic acid methyl ester (E6001). Quantitation of FAMEs was carried out with reference to the internal standard and by determining the response factors of individual FAMEs as described earlier [9]. Considering possible co-elution of the positional trans isomers [10], the values obtained for all the isomers for trans-octadecadienoic acid and trans-octadecatrienoic acid were individually pooled.

### Statistical analysis

Extraction of fats from the foods, derivatization to FAME and analysis on GC was carried out in triplicates. For ANOVA between the three types of snacks–perishable deep fried, non-perishable deep fried and the bakery products–for various parameters, Shapiro-Wilk’s normality test was first conducted. Since the test was negative individually for the datasets of SFA, MUFA, PUFA, TFA, and PUFA/SFA, One Way ANOVA on ranks (Kruskal-Wallis test), which is appropriate for unequal variance and sample size was performed followed by performing pairwise multiple comparisons using Dunn's method. As the data of fat content was normally distributed with equal variance as determined by Brown-Forsythe's test, Gabriel's post hoc test (suitable for equal variance and unequal sample sizes) was applied following ANOVA. All the tests were conducted at p ≤ 0.05. Principal Component Analysis was performed on the dataset of proportions of the four classes of fatty acids across the 75 samples. All statistical analyses were performed using SigmaPlot V13 except Gabriel's post hoc test which was performed using SPSS.

## Results and discussion

### Overall fatty acid profiles

**Fig 1:**
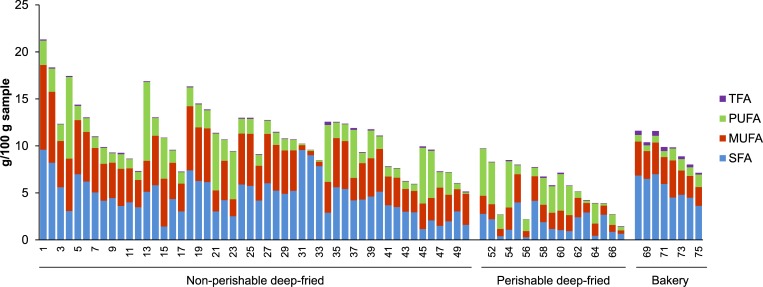
Concentrations (g/100 FW of food) of various classes of fatty acids–SFA, saturated fatty acids; MUFA, monounsaturated fatty acids; PUFA, polyunsaturated fatty acids; TFA, trans fatty acids–in the 75 Indian food samples analysed. Numbers on the X-axis represent sample numbers as detailed in Supplementary Table (S1 Table).

Seventy-five snack samples representing 33 snacks which were analysed for the fatty acid composition could be classified into three categories: perishable deep-fried (17 samples), non-perishable deep-fried (50 samples) and bakery (8 samples) (S1 Table). The classification as perishable or non-perishable was based on the fact that the former foods are supposed to be consumed on the same day of manufacturing; whereas, the latter ones have shelf life of at least a few weeks. The detected fatty acids were classified into four types: saturated (SFA), monounsaturated (MUFA), polyunsaturated (PUFA), and trans (TFA) fatty acids with SFA being the largest class (Fig 1, Tables 1 and 2). Eleven fatty acids viz., lauric (C12:0), myristic (C14:0), palmitic (C16:0), heptadecanoic (C17:0), stearic (C18:0), arachidic (C20:0), behenic (C22:0), palmitoleic (C16:1 n-7), oleic (C18:1 n-9), linoleic (C18:2 n-6), and linolenic acids (C18:3 n-3) were detected in all the samples (Tables 1 and 2, and S2 Table). The range of fatty acid concentrations considering all the samples together was 0.29–9.59 g/100 g FW (fresh weight) for SFA, 0.35–9.02 g/100 g for MUFA; 0.12–8.68 g/100 g for PUFA, and 0.001–0.51 g/100 g for TFA (Tables 1 and 2).

**Table 1:** Fatty acid composition (g/ 100 g FW of food samples) of the non-perishable deep fried food samples from India. Range of fatty acid concentration has been shown for multiple samples of the same foods. Details of samples are mentioned in Supplementary table (S1 Table).

Sample name	Gathiya	Potato Chips	Chakali	Sev	Fryums	Banana Chips	Maddar	Fried Bundi	Farsan	Shankar-pali	Farsan Papdi	Puri	Fried Noodles	Murukku	Papadi	Potato Sticks	Chiwda	Fafda
**Sample no.**	1–3	4–12	13–17	18–23	24–26	27–33	34	35	36	37–38	39	40–44	45	46	47	49	48	50
**No. of samples**	3	9	5	6	3	7	1	1	1	2	1	5	1	1	1	1	1	1
Octanoic acid	0.003–0.003	0.002–0.022	0–0.006	0.001–0.008	0–0.005	0.003–0.916	0.015	0.003	0.005	0.002–0.003	0.002	0.002–0.004	0.003	0.085	0.004	0.003	0.071	0.006
Decanoic acid	0.003–0.003	0–0.10	0–0.005	0.001–0.003	0.003–0.004	0.002–0.595	0.002	0.003	0.003	0.002–0.002	0.002	0.002–0.002		0.058	0.004	0.002	0.049	-
Lauric acid	0.030–0.033	0.006–0.080	0.001–0.056	0.002–0.033	0.018–0.023	0.02–4.777	0.006	0.025	0.031	0.006–0.024	0.022	0.011–0.023	0.001	0.42	0.008	0.015	0.398	0.003
Tridecanoic acid	-	-	-	-	-	0–0.005	-	-	-	-	-	-	-	-	-	-	-	-
Myristic acid	0.117–0.189	0.041–0.172	0.018–0.147	0.074–0.157	0.083–0.114	0.097–2.035	0.051	0.119	0.12	0.093–0.114	0.11	0.06–0.104	0.008	0.218	0.025	0.062	0.178	0.016
Pentadecanoic acid	0.006–0.009	0.003–0.007	0.001–0.006	0.004–0.007	0–0.005	0.001–0.006	0.004	0.006	0.005	0.004–0.005	0.005	0.003–0.005	0.002	0.005	0.004	0.003	0.001	0.003
Palmitic acid	4.846–8.41	2.308–5.974	0.998–5.032	2.125–6.45	3.652–5.181	0.81–5.22	2.183	4.861	4.703	3.591–3.722	3.974	2.538–4.476	0.725	0.838	0.874	2.597	0.783	1.056
Heptadecanoic acid	0.02–0.031	0.012–0.026	0.01–0.028	0.014–0.026	0.016–0.022	0.003–0.021	0.021	0.023	0.018	0.015–0.021	0.019	0.013–0.018	0.007	0.009	0.017	0.012	0.006	0.012
Stearic acid	0.524–0.844	0.312–0.650	0.275–0.52	0.255–0.67	0.375–0.529	0.247–0.563	0.505	0.512	0.499	0.387–0.421	0.436	0.281–0.455	0.317	0.362	0.254	0.311	0.28	0.39
Arachidic acid	0.022–0.034	0.002–0.025	0.012–0.025	0.014–0.027	0.014–0.022	0.004–0.023	0.017	0.024	0.021	0.016–0.02	0.02	0.011–0.019	0.011	0.01	0.048	0.012	0.038	0.016
Heneicosanoic acid	0.008–0.01	0.003–0.084	0.004–0.049	0–0.008	0.003–0.019	0–0.007	0.01	0.011		0.007–0.007	0.01	0.004–0.007	0.004	0.004	0.033	0.004	0.005	0.009
Behenic acid	0.008–0.011	0.004–0.074	0.006–0.045	0.008–0.017	0.005–0.008	0.001–0.007	0.052	0.007	0.007	0.005–0.013	0.01	0.004–0.006	0.054	0.044	0.137	0.004	0.114	0.06
Tricosanoic acid	0–0.005	0–0.004	0–0.006	0–0.006	0–0.003	0–0.004	0.006						0.004				0.004	0.007
Lignoceric acid	0.009–0.014	0–0.035	0.007–0.024	0.008–0.012	0–0.01	0.003–0.009	0.024	0.008	0.007	0.006–0.011	0.009	0.006–0.007	0.021	0.018	0.088	0.004	0.047	0.024
Myristoleic acid																		
cis-10-pentadecenoic acid																		
Palmitoleic acid	0.022–0.034	0.007–0.025	0.013–0.109	0.019–0.069	0.015–0.022	0.003–0.027	0.026	0.026	0.023	0.016–0.066	0.036	0.01–0.019	0.012	0.015	0.012	0.01	0.006	0.016
cis-10-heptadecenoic acid	0–0.003	0–0.003	0–0.004	0–0.006		0–0.003	0.004	0.004		0.003–0.006	0.005	0–0.003	0.003		0.005			0.004
cis-9-oleic acid	4.861–8.934	2.870–5.703	2.916–5.168	1.729–6.748	3.663–5.486	0.443–5.193	3.235	5.154	5.035	2.296–3.839	4.006	2.242–4.493	2.667	2.355	3.876	2.348	2.759	3.255
cis-11-eicosanoic acid	0.03–0.048	0.015–0.044	0.016–0.034	0.015–0.037	0.019–0.029	0.004–0.028	0.005	0.033	0.029	0.015–0.022	0.026	0.013–0.024	0.027	0.019	0.133	0.013	0.05	0.004
cis-11,14-eicosadienoic acid			0–0.004	0–0.004		0–0.002	0.004			0–0.003			0.003					0.005
Erucic acid			0–0.044			0–0.01						0–0.02			0.039		0.016	
Nervonic acid																		
Linoleic acid	1.68–2.488	0.837–8.614	1.177–8.334	1.833–5.905	1.124–1.548	0.093–1.331	5.907	1.565	1.713	1.066–5.027	2.819	0.69–1.31	5.909	5.003	1.602	0.579	2.336	0.04
γ-Linolenic acid		0–0.005																
α-Linolenic acid	0.079–0.114	0.044–0.124	0.039–0.09	0.099–0.135	0.044–0.049	0.014–0.069	0.107	0.1	0.094	0.037–0.039	0.088	0.028–0.045	0.026	0.016	0.022	0.024	0.021	0.121
cis-5,8,11,14,17-eicosapentaenoic acid										0–0.007	0.006							
cis-4,7,10,13,16,19-docosahexaenoic acid	0–0.019	0–0.018	0–0.026	0–0.03		0–0.024	0.04			0–0.04	0.027	0–0.018			0.028	0.013	0.025	0.006
Trans-octadecenoic acid	0.007–0.015	0.007–0.025	0.005–0.025	0.003–0.013	0.007–0.012	0.001–0.013	0.116	0.01	0.007	0.009–0.103	0.012	0.007–0.013	0.01	0.006	0.015	0.007	0.005	0.021
Trans- octadecadienoic acid	0.038–0.088	0.036–0.110	0.034–0.069	0.033–0.069	0.059–0.083	0.043–0.055	0.203	0.054	0.035	0.049–0.086	0.117	0.025–0.055	0.134	0.113	0.067	0.022	0.023	0.064
Trans- octadecatrienoic acid	0.007–0.016	0.003–0.022	0.007–0.009	0.005–0.012	0.005–0.014	0.007–0.009	0.021	0.010	0.008	0.004–0.009	0.012	0.003–0.010					0.004	0.003
Sum of SFA	6.538–10.52	4.009–7.912	2.364–6.741	2.640–8.336	4.588–6.832	5.838–10.5	3.832	6.532	6.354	5.147–5.221	5.554	3.872–6.063	2.094	3.007	2.433	3.963	2.912	2.539
Sum of MUFA	4.916–9.015	2.892–5.764	2.949–5.270	1.810–6.817	3.160–5.536	0.45–5.247	3.275	5.211	5.087	2.382–3.88	4.073	1.7–4.539	2.709	2.389	4.065	2.371	2.832	3.279
Sum of PUFA	1.778–2.601	0.889–8.684	0.039–0.082	1.638–5.088	1.049–1.596	0.117–1.314	6.054	1.665	1.806	1.105–5.114	2.94	0.538–1.355	5.939	5.019	1.652	0.616	2.383	0.173
Sum of TFA	0.052–0.114	0.055–0.142	0.039–0.082	0.037–0.091	0.066–0.109	0.001–0.077	0.339	0.071	0.049	0.067–0.193	0.141	0.028–0.077	0.144	0.119	0.081	0.034	0.027	0.088
Total	13.28–22.26	8.265–18.38	8.196–17.81	10.39–17.25	10.06–13.94	9.39–13.69	13.5	13.45	13.30	10.27–12.84	12.71	6.894–12.03	10.89	10.53	8.231	6.984	8.153	6.079

**Table 2:** Fatty acid compositions (g/ 100 g FW of food samples) of perishable deep-fried (sample no. 51–67) and bakery food samples (Sample no. 68–75) from India. Range of fatty acid concentration has been shown for multiple samples of the same foods. Details of samples are given in Supplementary Table (S1 Table).

Sample name	Bhaji	Batata Vada	Samosa	Pav Pattice	Medu Vada	Paratha	French fries	Jalebi	Fried Momo	Cream Roll	Khari	Butter Snack	Soyapuff	Cookies	Veg Puff
**Sample no.**	51–53	54–56	57–59	60	61–62	63	64	65	66–67	68–69	70	71	72	73	74–75
**No. of samples**	3	3	3	1	2	1	1	1	2	2	1	1	1	1	2
Octanoic acid	0.001–0.003	0.001–0.004	0.001–0.005	0.003	0.008–0.033	0.044	0.004	0.03	0.001–0.003	0.007–0.021	0.009		0.002	0.001	0.003–0.005
Decanoic acid	0–0.001	0–0.002	0–0.003		0.006–0.014	0.09		0.055	0.001–0.001	0.007–0.018	0.008	0.002	0.002	0.002	0.003–0.005
Lauric acid	0.001–0.006	0.001–0.012	0.003–0.019	0.003	0.054–0.116	0.112	0.001	0.074	0.002–0.007	0.083–0.212	0.086	0.022	0.021	0.015	0.026–0.052
Tridecanoic acid								0.003							
Myristic acid	0.003–0.082	0.003–0.085	0.021–0.082	0.011	0.053–0.067	0.469	0.003	0.366	0.012–0.013	0.148–0.179	0.15	0.106	0.095	0.087	0.077–0.085
Pentadecanoic acid	0.001–0.003	0.001–0.015	0.002–0.005	0.002	0.001–0.002	0.052	0.001	0.047		0.006–0.006	0.007	0.007	0.004	0.005	0.003–0.004
Palmitic acid	0.244–2.329	0.182–3.466	0.836–3.658	0.71	0.451–2.038	1.553	0.265	1.536	0.524–0.716	5.157–5.741	5.718	5.01	3.887	4.003	2.884–3.68
Heptadecanoic acid	0.003–0.017	0.002–0.016	0.006–0.015	0.008	0.008–0.009	0.051	0.004	0.054	0.004–0.004	0.021–0.024	0.023	0.019	0.016	0.017	0.012–0.016
Stearic acid	0.106–0.28	0.077–0.367	0.224–0.361	0.24	0.201–0.214	0.509	0.13	0.492	0.089–0.102	0.784–0.845	0.96	0.722	0.438	0.625	0.543–0.643
Arachidic acid	0.004–0.015	0.003–0.015	0.007–0.014	0.008	0.007–0.009	0.008	0.005	0.009	0.002–0.003	0.02–0.022	0.021	0.016	0.019	0.014	0.013–0.015
Heneicosanoic acid	0–0.003	0–0.004	0–0.017		0.004–0.004	0.006		0.007		0.005–0.005	0.003	0.037	0.005	0.015	
Behenic acid	0.012–0.017	0.005–0.047	0.006–0.026	0.035	0.003–0.034	0.011	0.021	0.008	0.006–0.008	0.006–0.007	0.006	0.005	0.008	0.008	0.01–0.013
Tricosanoic acid	0–0.004	0–0.005								0–0.003			0.003		
Lignoceric acid	0.007–0.008	0.005–0.018	0–0.005	0.015	0.003–0.013	0.005	0.009		0.003–0.004	0.007–0.008	0.007		0.008	0.007	0.007–0.007
Myristoleic acid						0.031		0.018							
cis-10-pentadecenoic acid						0.019		0.017							
Palmitoleic acid	0.004–0.065	0.003–0.013	0.008–0.012	0.01	0.007–0.009	0.086	0.005	0.071	0.002–0.003	0.012–0.014	0.013	0.017	0.017	0.011	0.009–0.009
cis-10-heptadecenoic acid		0–0.002	0–0.003	0.002		0.015		0.015		0–0.002			0.003		
cis-9-oleic acid	0.762–1.85	0.615–2.958	1.701–2.582	2.033	1.676–2.041	0.854	1.275	0.825	0.345–0.723	2.924–3.576	3.318	2.83	3.904	2.542	1.977–2.284
cis-11-eicosanoic acid	0.007–0.013	0.007–0.026	0.014–0.020	0.02	0.01–0.014	0.013	0.01	0.011	0.003–0.006	0.016–0.02	0.018	0.016	0.022	0.016	0.012–0.015
cis-11,14-eicosadienoic acid	0–0.003	0–0.003	0–0.004												
Erucic acid		0–0.051		0.003				0.015	0–0.003						
Nervonic acid		0–0.003													
Linoleic acid	1.461–4.93	0.933–4.779	0.873–2.761	3.778	0.549–3.074	0.208	2.122	0.175	0.373–1.037	0.605–0.695	0.706	0.618	1.201	1.067	0.9–1.321
γ-Linolenic acid															
α-Linolenic acid	0.049–0.094	0.026–0.087	0.032–0.475	0.117	0.026–0.118	0.044	0.019	0.022	0.011–0.073	0.015–0.017	0.022	0.028	0.047	0.133	0.02–0.022
cis-5,8,11,14,17-eicosapentaenoic acid			0–0.006	0.006				0.007							
cis-4,7,10,13,16,19-docosahexaenoic acid	0–0.013	0–0.05	0–0.023	0.006	0–0.002	0.01	0.008		0–0.004				0.014	0.025	
trans- octadecenoic	0–0.005	0–0.009	0.016–0.043	0.008	0.002–0.006	0.024	0.003	0.02	0.002–0.025	0.247–0.341	0.415	0.3	0.064	0.205	0.112–0.199
Trans-linoleic acid	0.031–0.039	0.017–0.098	0.038–0.085	0.111	0.020–0.051	0.022	0.033	0.022	0.029–0.020	0.062–0.090	0.091	0.123	0.051	0.073	0.059–0.095
Trans-linolenic acid	0.002–0.002	0.005–0.008	0.007–0.039	0.014	0.000–0.004	0	0	0.004	0.000–0.004	0.003–0.006	0.004	0	0.007	0.011	0.003–0.003
Sum of SFA	1.323–3.698	0.816–4.927	2.1–3.398	1.971	1.908–3.352	3.845	1.378	3.616	1.586–1.788	7.431–7.780	7.934	6.882	5.445	5.736	4.551–6.221
Sum of MUFA	0.773–1.928	0.646–2.987	1.722–1.844	2.069	1.697–2.06	1.018	1.289	0.971	0.354–0.733	2.950–3.611	3.35	2.863	3.945	2.569	1.999–2.308
Sum of PUFA	1.51–5.002	0.651–4.92	0.905–2.875	3.907	0.669–3.1	0.261	2.149	0.047	0.259–1.11	0.620–0.711	0.729	0.645	1.262	1.225	0.92–1.342
Sum of TFA	0.031–0.044	0.017–0.112	0.063–0.137	0.134	0.027–0.057	0.046	0.037	0.047	0.030–0.035	0.312–0.436	0.509	0.423	0.122	0.289	0.174–0.307
Total	3.637–10.67	3.137–9.42	6.747–8.672	8.080	6.109–6.653	5.17	4.853	4.837	2.373–3.666	11.32–12.54	12.52	10.81	10.78	9.818	8.067–8.737

**Fig 2:**
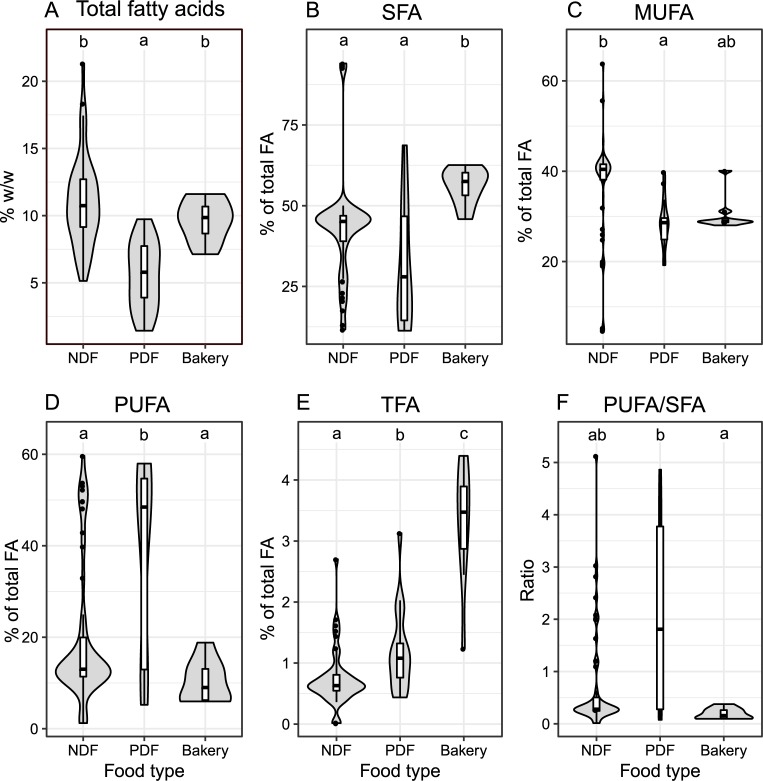
**Violin plot for comparisons of non-perishable deep-fried (NDF), perishable deep-fried (PDF), and bakery snacks for total fatty acid content (A), percent of SFA (B), MUFA (C), PUFA (D), and TFA in total fatty acids (E), and PUFA/SFA ratio (F).** Height of the box for each dataset on the x-axis denote the interquartile range, a thick horizontal line indicates median; whereas, upper and lower whiskers represent first and fourth quartile, respectively. Letters on the top of each plot indicate results of ANOVA and post-hoc tests detailed in Materials and Methods, wherein differing letters depict values significantly different from each other at p ≤ 0.05 within that plot.

Within the three food categories, average fat content (as sum of individual fatty acids) was significantly (*p*<0.05) higher for non-perishable deep-fried (11 g/100 g FW) and bakery (9.66 g/100 g FW) samples in comparison to perishable deep-fried products (5.3 g/100 g FW) (Fig 2). This could be due to more water loss during frying of the non-perishable deep-fried products, which is known to enhance the fat uptake. Such inverse correlation between moisture and fat content has been reported earlier for fried bread sticks [11]. High fat content in non-perishable deep fried snacks is also consistent with an earlier studies on Sev, a snack belonging to the same category, wherein incorporation of bacterial polysaccharides into the dough has been proposed as a strategy for minimizing the oil absorption [12].

Bakery products had significantly higher average proportion of SFA (56.1%) as well as TFA (3.26%) in total fatty acids as compared to both perishable (32.3% and 1.2%, respectively) and non-perishable deep fried categories (43% and 0.737%, respectively) (Fig 2). The higher proportion of TFA in bakery products as compared to other types of foods is in agreement with earlier reports on German [13] and Swiss [14] foods. Such high proportions of TFA as well as SFA in the bakery products are likely to be derived from the usage of margarine or bakery shortenings, both of which are partially hydrogenated vegetable oils (PHVO) [15]. The process of deodorization as well as repeated heating and frying of edible oils, especially those rich in PUFA, has also been reported to produce trans fatty acid isomers of oleic, linoleic and linolenic acids [3,16,17]. This might explain the significantly higher proportion (*p*< 0.05) of TFA in the perishable deep-fried samples which also contain significantly higher amounts of PUFA (38%) as compared to the non-perishable food samples (19.7%) and bakery foods (10.2%) (Fig 2). The average ratio of PUFA: SFA was also significantly higher for perishable deep-fried samples (average, 2.08; range, 0.0761–4.863) as compared to the bakery products (average, 0.19; range, 0.095–0.371) (Fig 2).

Similar observations were made when the data was analysed in a different way as follows. Within perishable deep-fried category, the highest proportion (68.8%) of samples contained PUFA as the dominant type of FA (Fig 1). On the other hand, for both non-perishable deep-fried and bakery categories, highest proportion of samples (74% and 100%, respectively) had SFA as the dominant type of FA (Fig 1). This suggests the SFA-rich oils could have been preferred for making bakery products as well as non-perishable fried foods while PUFA-rich oils could have been used for a majority of the perishable foods analysed in the present study.

### Saturated fatty acids

SFA was the most dominant class of fatty acids in about two thirds of the samples including all the bakery products (Fig 1). The highest range of SFA content (6.54–10.52 g/ 100 FW) was demonstrated by Gathiya (Sample 1–3), a nonperishable deep-fried snack (Table 1, Fig 1). On the other hand, the highest proportion of SFA in total fatty acids was found in three banana chips samples (samples 31–33) in the range of 92–94%, wherein lauric acid (C12:0) was the most dominant fatty acid followed by myristic acid (C14:0) (S2 Table). Half of the SFA were detected in all the samples with palmitic acid (C16:0) being the most dominant SFA in 72 of 75 samples (S2 Table). The average concentration and proportion in the total fatty acids of palmitic acid were 3.16 g/100 g FW and 31.7%; whereas, the highest and lowest concentration of 8.41 g/100 g FW and 0.18 g/100 g FW were detected in Gathiya (sample 1) and Batata Vada (sample 56), respectively (S2 and S3 Tables). Highest and lowest proportions of the palmitic acid in the total fatty acids were detected in Sample 71, Butter Snack (50.7%) and Sample 64, French fries (6.77%), respectively (Table 2). The dominance of SFA in the total fats and that of palmitic acid in SFA is consistent with numerous Indian and worldwide studies (For example, [5,10,18]. Dominance of lauric acid amongst all the fatty acids is not very commonly reported but has been found in some Chinese foods such as cake, cracker and wafer [19] and in a Cretan potato crisps sample [20]. Amongst cooking oils, coconut oil is reported to be highly rich in lauric acid [21].

### Monounsaturated fatty acids

The highest range of MUFA content (4.92–9.02 g/ 100 FW) was also demonstrated by Gathiya (Sample 1–3) (S1 Table, Fig 1). MUFA was also depicted as the most dominant class of fatty acids (range 42.7–63.9%) in only four samples: 10, Potato Chips; 15, Chakli; 47, Papadi; and 50, Fafda, all of which were non-perishable deep-fried sample (S2 Table). Most of the earlier studies on Indian foods have also indicated that MUFA were not the most dominant types of fatty acids in these foods [4,5,7,8]. However, few other studies have shown that groundnut, canola and olive oils [21–23], and some of the Cretan and Cypriot snacks [20] had highest levels of MUFA than any other fatty acids. Oleic acid (C18:1 n-9) was the fatty acids found in the highest concentration (8.93 g/100g in Gathiya, sample 1) and at highest proportion of total fatty acids (63.3% in Fafda, sample 50) in any single sample (S2 Table). Oleic acid was also the most dominant MUFA across all 75 samples (Tables 1 and 2). The next most dominant MUFA (cis-11-eicosanoic acid, C20:1 n-9) had relatively very low maximum concentration (0.13 g/100 FW in Papadi, sample 47) (Table 1). This observation is also similar to that reported in the above-mentioned studies.

### Polyunsaturated fatty acids

PUFA was the most dominant category of the fatty acids in 19 samples in the proportion of 40.7%-59.7% (Fig 1, S2 and S3 Tables). The highest range of PUFA concentration (0.89–8.68 g/100 g FW) was found in potato chips (sample 4–12) (Table 1). Linoleic acid (C18:2 n-6) was detected at the highest concentration (maximum 8.61 g/100 g FW, in Potato chips, sample 4) and highest proportion in total fatty acids (maximum 59.4% in Fried Noodles, sample 45) amongst all the PUFA in all the individual samples except Fafda (sample 50), where linolenic acid (C18:3 n-3) was the most dominant PUFA (Table 1, S2 Table). Both linoleic and linolenic acids were detected in all the samples (Tables 1 and 2). High levels of PUFA observed in several samples in this study are in contrast to some of the earlier studies. Very few foods studied by Gupta et al. (2016) and none of the foods analyzed by [7,8,10,18] have reported such high PUFA content in the foods. These results thus probably indicate recent consumer preference towards the PUFA rich sources such as sunflower and soybean oils over the conventional oils [24]. This prediction is also supported by the fact that in India, the imports of sunflower and soybean oils during last four years have increased by 133% and 108%, respectively, in contrast to merely 32% increase in the import of palmolein and 61% in all edible oils [25].

### Trans fatty acids

The highest concentration and proportion in total fatty acids of TFA (0.509 g/100 g FW, 4.4%) was found in a bakery product, Khari (sample 70) (Table 2). In India, the current limit of the TFA content imposed by Food Safety and Standards Authority of India (FSSAI) is 5% and they have recently launched a campaign which aims at reducing it to 2% by the year 2022 [26]. In the light of this fact, it is interesting to note that none of the samples analyzed in the present study had TFA content more than 5% of the total fats; whereas, all but one bakery products along with two perishable deep fried and one nonperishable deep fried samples had the TFA content exceeding 2% (S2 Table). Furthermore, the overall range of TFA content in the total fatty acid observed in the present study (0.016–4.4%) was much lower than the earlier reported ranges of 0.1–30% [5], 0.048–22.53% [7], and 1.9–53% [27] for Indian snacks. This is an indication of reducing trend in the TFA content in the Indian snacks which is further consistent with the reports from other countries including Costa-Rica [22] and Portugal [10].

Amongst the TFA, elaidic acid (trans-octadecenoic acid, C18:1trans n-9) was detected in the highest number of samples (73 of 75). The concentration and proportion in total fatty acids of elaidic acid detected in Khari, sample 70 (0.42 g/100 g FW, 3.58%), were higher than any other TFA in any sample (Tables 1 and 2, S2 Table). The dominance of elaidic acid amongst all the trans fatty acids has also been reported in earlier studies on fried Indian foods as well as foods from other countries (Bansal et al., 2009; Dias et al., 2015; Dorni et al., 2018; Fu et al., 2008; Reshma et al., 2012; Santos et al., 2015). Trans-octadecadienoic acid (C18:2 trans n-6) was found in 72 samples and was the TFA detected at the highest average proportion (0.64%) in the total fatty acids amongst all the TFA (S2 and S3 Tables). Indeed, in 61 samples, the levels of trans-octadecadienoic acid were higher than trans-octadecenoic acid (S2 and S3 Tables). Although a majority of the worldwide studies till now suggest elaidic acid as the most common TFA, some foods have been shown to have higher levels of trans-octadecadienoic acid than that of elaidic acid. These foods include uncooked palmolein, cooking oil and sunflower oil [16], most of the Costa Rican edible oils [22], and some of the Spanish [28][29], Brazilian [18], and Portuguese [30] snacks. The health effects of trans-octadecadienoic acid appear to be inadequately studied [31]; nonetheless, some studies indicate positive association of trans linoleic acids in the human erythrocytes with higher levels of LDL cholesterol [32]. The highest proportion of trans-octadecatrienoic acid isomers considered together in any sample was quite low (0.586% in Samosa, sample 58).

High variations in the quantities of total fatty acids and individual classes of fatty acids were observed within the groups of samples of similar foods (Tables 1 and 2, Fig 1). Although variation in the total fatty acid content could be because of varying extents of absorption of oils by these foods during frying, variation in the proportion of fatty acid classes is an indication of usage of different types of oils by different manufacturers of the similar foods. Various types of cooking oils in India have already been reported to have varying proportions of the fatty acids [21].

The ratio of omega-6/omega-3 fatty acids for all 75 samples ranged between 0.3–315 (Table 1 and S2 Table) with no significant difference between the three types of snacks (data not shown). Also, recent studies have suggested that there might not be a correlation between this ratio and its impact on human health [33,34].

### Principal component analysis

**Fig 3:**
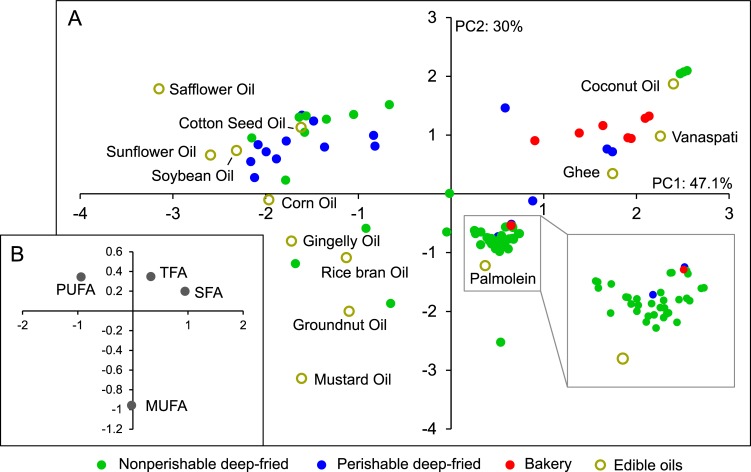
Principal component analysis of the content of various classes of fatty acids across 75 snack samples analysed in India. Data for the fatty acid content of various edible oils was obtained from Dorni et al (2018). (A) Score plot and (B) loading plot. Component loadings are detailed in Supplementary Table (S4 Table).

Considering the multidimensionality in the data, it was subjected to Principal Component Analysis (PCA) to depict the overall closeness of the foods samples to each other in terms of the proportions of various classes of fatty acids in them. The data of fatty acid composition of Indian edible oils obtained from [21] was also included in the input data for PCA to indirectly infer the oil usage based on the fatty acid profiles. The analysis resulted in three principal components which explained the whole variation in the data. Many samples clustered according to the food type across the score plot (Fig 3A) which was plotted considering first two principal components accountable for 77% of the variation. Seven of the eight bakery samples were present in quadrant I apparently due to high proportion of SFA and TFA which is further supported by the localization of these fatty acids in quadrant I of the loading plot (Fig 3B). Vanaspati (PHVO mostly made from palm oil and very commonly used in India), coconut oil, and Ghee (clarified butter), all of which also have high levels of SFA with the former one also having high concentrations of TFA [21] were placed very close to this cluster. Furthermore, TFA content of bakery samples and PHVO [21] were also similar with respect to dominance of elaidic acid. This suggests that PHVO such as Vanaspati or bakery shortening might have been used in the bakery samples analysed in the present study.

Placement of three banana chips samples (samples 31–33) which had the highest proportion of SFA in the total fat (92–94%) amongst all samples at the extreme end in the quadrant I very close to coconut oil which too had very high proportion of SFA [21], points out at a possibility of the usage of coconut oil for these samples. Highest and lowest proportions of MUFA and TFA, respectively, in Soypuff (sample 72) amongst all bakery samples justify its distant location from the cluster of other bakery samples on the score plot.

Eleven of 17 perishable deep-fried samples were clustered in quadrant II. High levels of PUFA in these perishable deep-fried foods explained the overlap of these categories in quadrant II of score and loading plots, respectively. The presence of sunflower, safflower, soyabean, and cotton seed oils, all having higher proportion of PUFA [21], close to this clusters could be a possible indication of presence of these oils in the foods in the quadrant II. Eight non-perishable deep-fried samples (samples 3, 4, 13, 21, 23, 37, 45, and 46) had 42.9% - 59.7% PUFA which was much higher than the average PUFA content (19.7%) for non-perishable deep-fried category (S3 Table). This justifies the presence of these samples close to the samples having high PUFA content in the quadrant I.

A majority of the non-perishable deep-fried samples (34 of 50) were clustered in quadrant IV. Indeed these samples along with two other samples formed the most compact cluster along with palmolein suggesting their similar fatty acid profiles. These samples had relatively higher proportion of MUFA similar to palmolein which supports their co-localization in the lower half of the score and loading plots (Fig 3). As the presence of polyunsaturated fatty acids can cause rancidity in the non-perishable fried food products [35], such clustering also indicates preference of the manufacturers towards oils such as palmolein which have lower proportion of PUFA over sunflower or soybean oils which are PUFA-rich [21] for enhancing the shelf life of non-perishable products.

To summarise, PCA was found out to be a very useful tool to identify similarity of the food samples to each other based on their fatty acid profiles. Furthermore, we were also able to correlate the samples to the commonly used edible oils in India to indirectly predict the oil usage. In a similar study published earlier [29], food samples were grouped according to their fatty acid profiles and the fats used in making these products were extrapolated by the factor analysis.

## Conclusion

The overall analysis in the present study indicates that the levels of TFA in the studied samples were lower than those reported earlier. However, some samples, especially, bakery products showed higher concentrations of TFA than the next lower target set for India. Bakery products and a majority of the non-perishable products also had high amounts of SFA. High amounts of PUFA detected in one quarter of the samples indicate a positive trend of usage of PUFA-rich oils. However, there has been reluctance amongst food manufacturers in India towards replacing SFA with PUFA and reducing TFA because of the issues related to cost and physical properties [36]. This fact underlines the importance of monitoring fatty acid profiles of the readymade foods.

## Supporting information

S1 Table Details of the samples analyzed in the present study. (XLSX)

S2 Table Concentrations of fatty acids (g/100 g FW) in 75 foods from India. Samples numbers refer to the foods detailed in S1 Table.
(XLSX)

S3 Table Proportion (%) of each fatty acid in the total fatty acid content in 75 foods from India. Samples numbers refer to the foods detailed in S1 Table.
(XLSX)

S4 Table Component scores of the PCA. Sample details can be found in S1 Table.
(XLSX)
